# Supplementary material for: Impacts of COVID-19-related service disruptions on TB incidence and deaths in Indonesia, Kyrgyzstan, Malawi, Mozambique, and Peru: Implications for national TB responses
Source: PLOS Glob Public Health. 2022 Mar 30;2(3):e0000219. doi: 10.1371/journal.pgph.0000219 (PMC10021439; doi:10.1371/journal.pgph.0000219)
Supplement: S1 Appendix — (DOCX) [file pgph.0000219.s001.docx]

**Impacts of COVID-19-related service disruptions on TB incidence and deaths in Indonesia, Kyrgyzstan, Malawi, Mozambique, and Peru: Implications for national TB responses**

Martin-Hughes R, Vu L, Cheikh N, Kelly SL, Fraser-Hurt N, Shubber Z, Manhiça I, Mbendera K, Girma B, Pambudi I, Ríos J, Elmira A, Harimurti P, Hafez R, Garcia JNB, Palmer T, Roberts A, Gorgens M, Wilson D

**Supplementary Appendix**

Levels of disruption given as values relative to 2019 status quo values for rates of TB diagnosis, treatment initiation, treatment completion, BCG vaccination, ART coverage, and HIV prevention. The uncertainty range between best-case and worse-case is typically a 10% range on reported levels of disruption in the national data for each parameter to reflect variation between reported numbers in quarters for reasons other than COVID-19-related service disruptions.

**Indonesia**

| Time period | Scenario | DS-TB diagnosis | DR-TB diagnosis | DS-TB treatment initiation | DR-TB treatment initiation | DS-TB treatment completion | DR-TB treatment completion | BCG vaccination | ART coverage | TB preventive therapy |
| --- | --- | --- | --- | --- | --- | --- | --- | --- | --- | --- |
| Q1 2020 | Status quo | 1 | 1 | 1 | 1 | 1 | 1 | 1 | 1 | 1 |
| Q2 2020 | Estimated best-case | 0.62 | 0.47 | 0.91 | 0.83 | 0.84 | 0.94 | 0.46 | 0.75 | 0.50 |
| Q2 2020 | Estimated worse-case | 0.54 | 0.35 | 0.89 | 0.79 | 0.80 | 0.50 | 0.34 | 0.25 | 0.17 |
| Q3 2020 | Estimated best-case | 0.67 | 0.51 | 0.91 | 0.83 | 0.84 | 0.94 | 0.46 | 0.75 | 0.59 |
| Q3 2020 | Estimated worse-case | 0.59 | 0.40 | 0.89 | 0.79 | 0.80 | 0.50 | 0.34 | 0.25 | 0.20 |
| Q4 2020 | Estimated best-case | 0.57 | 0.47 | 0.91 | 0.83 | 0.84 | 0.94 | 0.46 | 0.75 | 0.55 |
| Q4 2020 | Estimated worse-case | 0.47 | 0.35 | 0.89 | 0.79 | 0.80 | 0.50 | 0.34 | 0.25 | 0.19 |
| Q1 2021 | Estimated best-case | 0.52 | 0.41 | 0.90 | 0.81 | 0.82 | 0.72 | 0.40 | 0.75 | 0.37 |
| Q1 2021 | Estimated worse-case | 0.43 | 0.31 | 0.88 | 0.77 | 0.78 | 0.39 | 0.30 | 0.25 | 0.13 |
| Q2 2021 | Estimated best-case | 0.57 | 0.47 | 0.91 | 0.83 | 0.84 | 0.94 | 0.46 | 0.75 | 0.55 |
| Q2 2021 | Estimated worse-case | 0.47 | 0.35 | 0.89 | 0.79 | 0.80 | 0.50 | 0.34 | 0.25 | 0.19 |
| Q3 2021 – Q2 2022 | Estimated best-case | 0.67 | 0.51 | 0.91 | 0.83 | 0.84 | 0.94 | 0.46 | 0.75 | 0.59 |
| Q3 2022 – Q4 2022 |  | 1 | 1 | 1 | 1 | 1 | 1 | 1 | 1 | 1 |
| Q3 2021 – Q4 2022 | Estimated worse-case | 0.47 | 0.35 | 0.89 | 0.79 | 0.80 | 0.50 | 0.34 | 0.25 | 0.17 |
| Q3 2021 – Q2 2022 | Catch-up best-case | 1.07 | 1.01 | 1.11 | 1.00 | 1.00 | 1.00 | 1.00 | 1.00 | 1.59 |
| Q3 2022 – Q4 2022 |  | 1.40 | 1.50 | 1.20 | 1.10 | 1.10 | 1.05 | 1.55 | 1.00 | 2.00 |
| Q3 2021 – Q4 2022 | Catch-up worse-case | 0.87 | 0.85 | 1.09 | 0.96 | 0.96 | 0.56 | 0.88 | 0.50 | 1.17 |

**Kyrgyzstan**

| Time period | Scenario | DS-TB diagnosis | DR-TB diagnosis | DS-TB treatment initiation | DR-TB treatment initiation | DS-TB treatment completion | DR-TB treatment completion | BCG vaccination | ART coverage | TB preventive therapy |
| --- | --- | --- | --- | --- | --- | --- | --- | --- | --- | --- |
| Q1 2020 | Status quo | 1 | 1 | 1 | 1 | 1 | 1 | 1 | N/A | 1 |
| Q2 2020 | Estimated best-case | 0.577 | 0.478 | 1 | 1 | 1 | 1 | 1 |  | 0.55 |
| Q2 2020 | Estimated worse-case | 0.483 | 0.362 | 1 | 1 | 1 | 1 | 1 |  | 0.45 |
| Q3 2020 | Estimated best-case | 0.649 | 0.523 | 1 | 1 | 1 | 1 | 1 |  | 0.91 |
| Q3 2020 | Estimated worse-case | 0.571 | 0.417 | 1 | 1 | 1 | 1 | 1 |  | 0.89 |
| Q4 2020 | Estimated best-case | 0.658 | 0.712 | 1 | 1 | 1 | 1 | 1 |  | 0.91 |
| Q4 2020 | Estimated worse-case | 0.483 | 0.417 | 0.9 | 0.9 | 0.9 | 0.9 | 0.9 |  | 0.78 |
| Q1 2021 | Estimated best-case | 0.658 | 0.712 | 1 | 1 | 1 | 1 | 1 |  | 0.91 |
| Q1 2021 | Estimated worse-case | 0.483 | 0.417 | 0.9 | 0.9 | 0.9 | 0.9 | 0.9 |  | 0.78 |
| Q2 2021 | Estimated best-case | 0.658 | 0.712 | 1 | 1 | 1 | 1 | 1 |  | 0.91 |
| Q2 2021 | Estimated worse-case | 0.483 | 0.417 | 0.9 | 0.9 | 0.9 | 0.9 | 0.9 |  | 0.78 |
| Q3 2021 – Q2 2022 | Estimated best-case | 0.8 | 0.8 | 1 | 1 | 1 | 1 | 1 |  | 1 |
| Q3 2022 – Q4 2022 |  | 1 | 1 | 1 | 1 | 1 | 1 | 1 |  | 1 |
| Q3 2021 – Q4 2022 | Estimated worse-case | 0.483 | 0.417 | 1 | 1 | 1 | 1 | 1 |  | 0.78 |
| Q3 2021 – Q2 2022 | Catch-up best-case | 1 | 1.2 | 1.1 | 1.1 | 1.1 | 1.1 | 1 |  | 1.3 |
| Q3 2022 – Q4 2022 |  | 1.2 | 1.4 | 1.1 | 1.1 | 1.1 | 1.1 | 1 |  | 1.3 |
| Q3 2021 – Q4 2022 | Catch-up worse-case | 0.683 | 0.817 | 1.1 | 1.1 | 1.1 | 1.1 | 1 |  | 1.08 |

**Malawi – Blantyre**

* TB preventive therapy 1.1 as status quo held from Q3 2020 onward

| Time period | Scenario | DS-TB diagnosis | DR-TB diagnosis | DS-TB treatment initiation | DR-TB treatment initiation | DS-TB treatment completion | DR-TB treatment completion | BCG vaccination | ART coverage | * TB preventive therapy |
| --- | --- | --- | --- | --- | --- | --- | --- | --- | --- | --- |
| Q1 2020 | Status quo | 1 | 1 | 1 | 1 | 1 | 1 | 1 | 1 | 1 |
| Q2 2020 | Estimated best-case | 0.658 | 0.658 | 1 | 1 | 1 | 1 | 1 | 1 | 1 |
| Q2 2020 | Estimated worse-case | 0.582 | 0.582 | 1 | 1 | 1 | 1 | 1 | 1 | 1 |
| Q3 2020 | Estimated best-case | 0.8 | 0.8 | 1 | 1 | 1 | 1 | 1 | 1 | 1 |
| Q3 2020 | Estimated worse-case | 0.6 | 0.5 | 0.9 | 0.9 | 0.9 | 0.9 | 1 | 0.8 | 0.8 |
| Q4 2020 | Estimated best-case | 1 | 1 | 1 | 1 | 1 | 1 | 1 | 1 | 1 |
| Q4 2020 | Estimated worse-case | 0.8 | 0.8 | 0.9 | 0.9 | 0.9 | 0.9 | 1 | 1 | 1 |
| Q1 2021 | Estimated best-case | 0.658 | 0.6 | 1 | 1 | 1 | 1 | 1 | 1 | 1 |
| Q1 2021 | Estimated worse-case | 0.582 | 0.4 | 0.75 | 0.75 | 0.75 | 0.75 | 0.75 | 0.75 | 0.75 |
| Q2 2021 | Estimated best-case | 0.8 | 1 | 1 | 1 | 1 | 1 | 1 | 1 | 1 |
| Q2 2021 | Estimated worse-case | 0.6 | 0.8 | 0.9 | 0.9 | 0.9 | 0.9 | 1 | 1 | 1 |
| Q3 2021 – Q2 2022 | Estimated best-case | 0.9 | 0.9 | 1 | 1 | 1 | 1 | 1 | 1 | 1 |
| Q3 2022 – Q4 2022 |  | 1 | 1 | 1 | 1 | 1 | 1 | 1 | 1 | 1 |
| Q3 2021 – Q4 2022 | Estimated worse-case | 0.6 | 0.6 | 0.9 | 0.9 | 0.9 | 0.9 | 0.9 | 0.9 | 0.9 |
| Q3 2021 – Q2 2022 | Catch-up best-case | 1.1 | 1.1 | 1 | 1 | 1 | 1 | 1 | 1 | 1.1 |
| Q3 2022 – Q4 2022 |  | 1.2 | 1.2 | 1 | 1 | 1 | 1 | 1 | 1 | 1.2 |
| Q3 2021 – Q4 2022 | Catch-up worse-case | 0.9 | 0.9 | 0.9 | 0.9 | 0.9 | 0.9 | 0.9 | 0.9 | 1 |

**Malawi – Lilongwe**

* TB preventive therapy 1.1 as status quo held from Q3 2020 onward

| Time period | Scenario | DS-TB diagnosis | DR-TB diagnosis | DS-TB treatment initiation | DR-TB treatment initiation | DS-TB treatment completion | DR-TB treatment completion | BCG vaccination | ART coverage | * TB preventive therapy |
| --- | --- | --- | --- | --- | --- | --- | --- | --- | --- | --- |
| Q1 2020 | Status quo | 1 | 1 | 1 | 1 | 1 | 1 | 1 | 1 | 1 |
| Q2 2020 | Estimated best-case | 0.82 | 0.82 | 1 | 1 | 1 | 1 | 1 | 1 | 1 |
| Q2 2020 | Estimated worse-case | 0.78 | 0.78 | 1 | 1 | 1 | 1 | 1 | 1 | 1 |
| Q3 2020 | Estimated best-case | 0.9 | 0.9 | 1 | 1 | 1 | 1 | 1 | 1 | 1 |
| Q3 2020 | Estimated worse-case | 0.8 | 0.5 | 0.9 | 0.9 | 0.9 | 0.9 | 1 | 0.8 | 0.8 |
| Q4 2020 | Estimated best-case | 1 | 1 | 1 | 1 | 1 | 1 | 1 | 1 | 1 |
| Q4 2020 | Estimated worse-case | 0.8 | 0.9 | 0.9 | 0.9 | 0.9 | 0.9 | 1 | 1 | 1 |
| Q1 2021 | Estimated best-case | 0.82 | 0.7 | 1 | 1 | 1 | 1 | 1 | 1 | 1 |
| Q1 2021 | Estimated worse-case | 0.78 | 0.4 | 0.75 | 0.75 | 0.75 | 0.75 | 0.75 | 0.75 | 0.75 |
| Q2 2021 | Estimated best-case | 0.8 | 1 | 1 | 1 | 1 | 1 | 1 | 1 | 1 |
| Q2 2021 | Estimated worse-case | 0.6 | 0.9 | 0.9 | 0.9 | 0.9 | 0.9 | 1 | 1 | 1 |
| Q3 2021 – Q2 2022 | Estimated best-case | 1 | 1 | 1 | 1 | 1 | 1 | 1 | 1 | 1 |
| Q3 2022 – Q4 2022 |  | 1 | 1 | 1 | 1 | 1 | 1 | 1 | 1 | 1 |
| Q3 2021 – Q4 2022 | Estimated worse-case | 0.6 | 0.6 | 0.9 | 0.9 | 0.9 | 0.9 | 0.9 | 0.9 | 0.9 |
| Q3 2021 – Q2 2022 | Catch-up best-case | 1.2 | 1.2 | 1 | 1 | 1 | 1 | 1 | 1 | 1.1 |
| Q3 2022 – Q4 2022 |  | 1.2 | 1.2 | 1 | 1 | 1 | 1 | 1 | 1 | 1.2 |
| Q3 2021 – Q4 2022 | Catch-up worse-case | 0.8 | 0.8 | 0.9 | 0.9 | 0.9 | 0.9 | 0.9 | 0.9 | 1 |

**Malawi – Mzimba**

* TB preventive therapy 1.1 as status quo held from Q3 2020 onward

| Time period | Scenario | DS-TB diagnosis | DR-TB diagnosis | DS-TB treatment initiation | DR-TB treatment initiation | DS-TB treatment completion | DR-TB treatment completion | BCG vaccination | ART coverage | * TB preventive therapy |
| --- | --- | --- | --- | --- | --- | --- | --- | --- | --- | --- |
| Q1 2020 | Status quo | 1 | 1 | 1 | 1 | 1 | 1 | 1 | 1 | 1 |
| Q2 2020 | Estimated best-case | 0.92 | 0.92 | 1 | 1 | 1 | 1 | 1 | 1 | 1 |
| Q2 2020 | Estimated worse-case | 0.78 | 0.78 | 1 | 1 | 1 | 1 | 1 | 1 | 1 |
| Q3 2020 | Estimated best-case | 0.9 | 0.8 | 1 | 1 | 1 | 1 | 1 | 1 | 1 |
| Q3 2020 | Estimated worse-case | 0.7 | 0.5 | 0.9 | 0.9 | 0.9 | 0.9 | 1 | 0.8 | 0.8 |
| Q4 2020 | Estimated best-case | 1 | 1 | 1 | 1 | 1 | 1 | 1 | 1 | 1 |
| Q4 2020 | Estimated worse-case | 0.8 | 0.9 | 0.9 | 0.9 | 0.9 | 0.9 | 1 | 1 | 1 |
| Q1 2021 | Estimated best-case | 0.83 | 0.7 | 1 | 1 | 1 | 1 | 1 | 1 | 1 |
| Q1 2021 | Estimated worse-case | 0.67 | 0.4 | 0.75 | 0.75 | 0.75 | 0.75 | 0.75 | 0.75 | 0.75 |
| Q2 2021 | Estimated best-case | 0.8 | 1 | 1 | 1 | 1 | 1 | 1 | 1 | 1 |
| Q2 2021 | Estimated worse-case | 0.7 | 0.9 | 0.9 | 0.9 | 0.9 | 0.9 | 1 | 1 | 1 |
| Q3 2021 – Q2 2022 | Estimated best-case | 0.9 | 0.9 | 1 | 1 | 1 | 1 | 1 | 1 | 1 |
| Q3 2022 – Q4 2022 |  | 1 | 1 | 1 | 1 | 1 | 1 | 1 | 1 | 1 |
| Q3 2021 – Q4 2022 | Estimated worse-case | 0.7 | 0.7 | 0.9 | 0.9 | 0.9 | 0.9 | 0.9 | 0.9 | 0.9 |
| Q3 2021 – Q2 2022 | Catch-up best-case | 1.1 | 1.1 | 1 | 1 | 1 | 1 | 1 | 1 | 1.1 |
| Q3 2022 – Q4 2022 |  | 1.2 | 1.2 | 1 | 1 | 1 | 1 | 1 | 1 | 1.2 |
| Q3 2021 – Q4 2022 | Catch-up worse-case | 0.9 | 0.9 | 0.9 | 0.9 | 0.9 | 0.9 | 0.9 | 0.9 | 1 |

**Mozambique**

*DS-TB diagnosis 1.12 and DR-TB diagnosis 1.2 as status quo held from Q2 2020 onward

| Time period | Scenario | * DS-TB diagnosis | * DR-TB diagnosis | DS-TB treatment initiation | DR-TB treatment initiation | DS-TB treatment completion | DR-TB treatment completion | BCG vaccination | ART coverage | TB preventive therapy |
| --- | --- | --- | --- | --- | --- | --- | --- | --- | --- | --- |
| Q1 2020 | Status quo | 1 | 1 | 1 | 1 | 1 | 1 | 1 | 1 | 1 |
| Q2 2020 | Estimated best-case | 1.00912 | 1.1 | 1 | 1 | 1 | 1 | 1 | 0.96 | 0.91 |
| Q2 2020 | Estimated worse-case | 0.98448 | 1 | 1 | 1 | 1 | 1 | 1 | 0.96 | 0.89 |
| Q3 2020 | Estimated best-case | 1 | 1.1 | 1 | 1 | 1 | 1 | 1 | 1 | 1 |
| Q3 2020 | Estimated worse-case | 0.89 | 0.88 | 0.9 | 0.9 | 0.9 | 0.9 | 1 | 0.76 | 0.9 |
| Q4 2020 | Estimated best-case | 1 | 1.1 | 1 | 1 | 1 | 1 | 1 | 1 | 1 |
| Q4 2020 | Estimated worse-case | 0.89 | 0.88 | 0.9 | 0.9 | 0.9 | 0.9 | 1 | 0.76 | 0.9 |
| Q1 2021 | Estimated best-case | 1.00912 | 1.1 | 1 | 1 | 1 | 1 | 1 | 0.89 | 1 |
| Q1 2021 | Estimated worse-case | 0.98448 | 0.75 | 0.75 | 0.75 | 0.75 | 0.75 | 0.75 | 0.75 | 0.75 |
| Q2 2021 | Estimated best-case | 1 | 1.1 | 1 | 1 | 1 | 1 | 1 | 1 | 1 |
| Q2 2021 | Estimated worse-case | 0.89 | 0.88 | 0.9 | 0.9 | 0.9 | 0.9 | 1 | 0.76 | 0.9 |
| Q3 2021 – Q4 2022 | Estimated best-case | 1.12 | 1.2 | 1 | 1 | 1 | 1 | 1 | 1 | 1 |
| Q3 2021 – Q4 2022 | Estimated worse-case | 0.89 | 0.89 | 0.9 | 0.9 | 0.9 | 0.9 | 0.9 | 0.9 | 0.9 |
| Q3 2021 – Q2 2022 | Catch-up best-case | 1.2 | 1.2 | 1 | 1 | 1 | 1 | 1 | 1 | 1.1 |
| Q3 2022 – Q4 2022 |  | 1.32 | 1.4 | 1 | 1 | 1 | 1 | 1 | 1 | 1.1 |
| Q3 2021 – Q4 2022 | Catch-up worse-case | 1.09 | 1.09 | 0.9 | 0.9 | 0.9 | 0.9 | 0.9 | 0.9 | 1 |

**Peru**

* XDR-TB diagnosis not disrupted; TB diagnosis differentiated by age

** Reduction of TB transmission assumed to scale linearly from 1 to 0.8 over 24 months from 1 January 2023 to 31 December 2024 then held constant at 0.8 as status quo from Q1 2025 onward. In the catch-up scenarios, this reduction in TB transmission from 1 to 0.8 occurs 18-months earlier, from 1 July 2021 until 30 June 2023 then held constant at 0.8 from Q3 2023 onward

| Time period | Scenario | DS-TB diagnosis (children 0-17) | DS-TB diagnosis (adults) | DR-TB diagnosis (children 0-17)* | DR-TB diagnosis (adults)* | DS-TB treatment initiation | DR-TB treatment initiation | DS-TB treatment completion | DR-TB treatment completion | BCG vaccination | TB preventive therapy | TB transmission ** |
| --- | --- | --- | --- | --- | --- | --- | --- | --- | --- | --- | --- | --- |
| Q4 2019 | Status quo | 1 | 1 | 1 | 1 | 1 | 1 | 1 | 1 | 1 | 1 | 1 |
| Q1 2020 | Reported | 0.94 | 0.93 | 0.93 | 0.94 | 1 | 1 | 1 | 1 | 0.94 | 0.78 | 1 |
| Q2 2020 | Estimated best-case | 0.424 | 0.541 | 0.424 | 0.541 | 1 | 1 | 1 | 1 | 0.424 | 0.379 | 1 |
| Q2 2020 | Estimated worse-case | 0.296 | 0.439 | 0.296 | 0.439 | 1 | 1 | 1 | 1 | 0.296 | 0.241 | 1 |
| Q3 2020 | Estimated best-case | 0.496 | 0.721 | 0.496 | 0.721 | 1 | 1 | 1 | 1 | 0.712 | 0.505 | 1 |
| Q3 2020 | Estimated worse-case | 0.384 | 0.659 | 0.384 | 0.659 | 1 | 1 | 1 | 1 | 0.648 | 0.395 | 1 |
| Q4 2020 | Estimated best-case | 0.604 | 0.865 | 0.604 | 0.865 | 1 | 1 | 1 | 1 | 0.838 | 0.613 | 1 |
| Q4 2020 | Estimated worse-case | 0.516 | 0.835 | 0.516 | 0.835 | 1 | 1 | 1 | 1 | 0.802 | 0.527 | 1 |
| Q1 2021 | Estimated best-case | 0.424 | 0.541 | 0.424 | 0.541 | 1 | 1 | 1 | 1 | 0.424 | 0.379 | 1 |
| Q1 2021 | Estimated worse-case | 0.296 | 0.439 | 0.296 | 0.439 | 1 | 1 | 1 | 1 | 0.296 | 0.241 | 1 |
| Q2 2021 | Estimated best-case | 0.496 | 0.721 | 0.496 | 0.721 | 1 | 1 | 1 | 1 | 0.712 | 0.505 | 1 |
| Q2 2021 | Estimated worse-case | 0.384 | 0.659 | 0.384 | 0.659 | 1 | 1 | 1 | 1 | 0.648 | 0.395 | 1 |
| Q3 2021 – Q2 2022 | Estimated best-case | 0.874 | 0.930 | 0.874 | 0.930 | 1 | 1 | 1 | 1 | 0.928 | 0.876 | 1 |
| Q3 2022 – Q4 2022 |  | 1 | 1 | 1 | 1 | 1 | 1 | 1 | 1 | 1 | 1 | 1 |
| Q3 2021 – Q4 2022 | Estimated worse-case | 0.479 | 0.672 | 0.479 | 0.672 | 1 | 1 | 1 | 1 | 0.615 | 0.467 | 1 |
| Q3 2021 – Q2 2022 | Catch-up best-case | 0.874 | 0.930 | 0.874 | 0.930 | 1 | 1.012 | 1.062 | 1 | 0.928 | 4.013 | 1 → 0.85 ** |
| Q3 2022 – Q4 2022 |  | 1 | 1 | 1 | 1 | 1 | 1.012 | 1.062 | 1 | 1 | 4.137 | 1 → 0.85 ** |
| Q3 2021 – Q4 2022 | Catch-up worse-case | 0.479 | 0.672 | 0.479 | 0.672 | 1 | 1.012 | 1.062 | 1 | 0.615 | 3.603 | 1 → 0.85 ** |
