# Supplementary material for: Impacts of COVID-19-related service disruptions on TB incidence and deaths in Indonesia, Kyrgyzstan, Malawi, Mozambique, and Peru: Implications for national TB responses
Source: PLOS Glob Public Health. 2022 Mar 30;2(3):e0000219. doi: 10.1371/journal.pgph.0000219 (PMC10021439; doi:10.1371/journal.pgph.0000219)
Supplement: Attachment — Submitted filename: Response to reviewers.docx [file pgph.0000219.s002.docx]

# Response to reviewers

We would like to thank the reviewers for their time. Responses are below (line numbers refer to tracked changes version).

**Reviewer #1:**

**1. The authors use mathematical modelling to explore the impact of COVID-19-related disruptions on TB incidence and deaths in 5 high TB burden countries. Importantly, the modelling has been conducted in conjunction with the National TB Programmes, and should be useful to decision-makers in these countries. I do think this is an interesting piece of work, and is particularly important to highlight the variability of the impact of COVID-19 on TB by setting.**

We appreciate this feedback.

**2. I appreciate that the timing has not been unhelpful, but there have been some major reports in the last month or two which should be included in your introduction and discussion. In particular, the WHO 2021 GTB report (**[**https://www.who.int/publications/i/item/9789240037021**](https://www.who.int/publications/i/item/9789240037021)**) and reports from the Global Fund (**[**https://www.theglobalfund.org/en/results/**](https://www.theglobalfund.org/en/results/)**) and Stop TB Partnership (**[**http://www.stoptb.org/news/stories/2021/ns21_035.html**](http://www.stoptb.org/news/stories/2021/ns21_035.html)**). There are also some broad reviews that might help helpful here (**[**https://doi.org/10.1183/13993003.01786-2021**](https://doi.org/10.1183/13993003.01786-2021) **and** [**https://www.ncbi.nlm.nih.gov/pmc/articles/PMC8171247**](https://www.ncbi.nlm.nih.gov/pmc/articles/PMC8171247)**). Where possible I feel it would also be better to see a comparison to data in your countries of interest rather than in e.g. India and Nigeria, as you currently have.**

**3. I would have like to have seen a more explicit comparison to the other modelling studies (they should have been cited, and separate issues in each highlighted) to help non-experts to understand the implications of these findings in comparison to other available evidence. For example, transmission and vulnerability (see** [**https://pubmed.ncbi.nlm.nih.gov/32513784/**](https://pubmed.ncbi.nlm.nih.gov/32513784/) **and** [**https://www.ncbi.nlm.nih.gov/pmc/articles/PMC7348601/**](https://www.ncbi.nlm.nih.gov/pmc/articles/PMC7348601/)**), have previously been considered, as well as vaccines alone (**[**https://www.mdpi.com/2076-393X/9/11/1228**](https://www.mdpi.com/2076-393X/9/11/1228)**).**

Thank you for these suggested references, which were primarily released during the submission process. We agree that inclusion of these latest references adds to the context of this paper, and have added these references throughout the Introduction (lines 55 – 95), as well as additional context of the data gaps that this study responds to.

**4. Although you mention the importance of overcrowding, you do consider changing transmission in Peru, but don’t discuss this in the text. Exploring this within your model should be comparatively easy? In addition, as mentioned above the recent WHO report has come out and this includes modelling in Indonesia and Peru, although details on the methodology are light. Overall it would also have been good to compare your results to these in the discussion, and to comment on why they may differ.**

We have added an additional description of the methodology behind changing transmission in Peru as part of the epidemiological inputs and Limitations (lines 313 – 329) to better explain this and other important assumptions around TB transmission.

We would also like to add more comparison to the modelling results presented by the WHO for Indonesia and Peru in the latest Global TB report released during submission of this work, but without either the raw data or an explanation of the methodology/assumptions it is hard to compare the results. The peak increase in infections/deaths appears to be higher in the Global TB report but we have plotted annual values rather than continuous values so the outputs may be consistent.

**5. I found it difficult to think through the model methods and evaluate the assumptions. It would have been helpful to see some form of parameter table (as in the appendix) in the main text, but I appreciate that was particularly large. Perhaps a colour-shaded version of the appendix tables would have been helpful, to help give an overview of the various impacts? Although you do include the parameter table, because the data is owned by the NTP that is not included here. It does make it quite difficult to evaluate the scenarios you have produced, but I appreciate that there are proprietary concerns that make it hard to resolve.**

The full model inputs are a little large for the main text, but we have implemented the excellent suggestion of colour-coding the appendix tables to give a clearer visual of the impacts. (S1 appendix)

**6. I think it would have been helpful to compare and contrast the impact on incidence vs deaths, including how these compare to the disruptions. For example, Mozambique experienced some of the least covid disruptions, yet had the largest increase in deaths (in worst case scenario). It also had about average increase in incidence. I think aspects such as that needed discussion.**

We have added some additional discussion to explain why the worse-case values for Mozambique and Malawi are higher but the actual estimate is likely closer to the best-case, in conjunction with additional details in the methodology (lines 278 – 286). The worse-case scenarios were based on extremely high uncertainty at the end of 2020 when both countries had not experienced the kinds of disruptions seen elsewhere but were concerned about the possibility. As the second and much larger wave of COVID-19 passed in early 2021 in both countries without that level of disruption, the worse-case scenario was considered far less likely.

**7. The figure axes legends were quite difficult to read, I would suggest including this information in the main text instead.**

We think the legends may have been unclear in the preview version of figures and hope that this concern will be addressed by the higher-quality final images.

**8. In the supporting material table, the catch-up scenario in Indonesia in particular seemed ambitious, particularly given they are currently in the worst-case scenario. Was this driven by the NTP? I also see that you have a very low reduction in transmission in Peru, but not elsewhere. This might be worth discussing.**

We have added to the methods that the “Modelled levels of disruption after 1 July 2021 were based on NTP best-case and worse-case estimates of the severity and duration of disruptions given prospective scenarios of low or severe external health system pressures due to COVID-19, rather than based on relative likelihood. Further qualitative evaluation of progress within this uncertainty interval was conducted following the modelling.” (lines 145 – 154)

We have also added some extra discussion of the results for Indonesia to clarify after the worsening of the epidemic in line with the worse-case projection, the NTP considered the implementation of the catch-up strategy to have limited feasibility as we concluded the modelling phase and evaluated the ongoing (and worsening at that time until August 2021) situation. (lines 292 – 305)

Reviewer #2:

**1. Methods**

**- In Table 1, “lower and upper bounds of uncertainty intervals for levels of disruption”: it’s unclear to me what these uncertainty intervals are. Are they the “low” and “severe” disruption levels from the supporting tables in the Appendix?**

**Table 1 is very important and I’d like this to be explained more clearly.**

We have added some additional explanation of Table 1 to the Model Inputs (lines 145 – 154). The lower and upper bounds of the uncertainty interval from 1 April 2020 until 30 June 2021 do correspond to the low and severe disruption scenarios in the Supplementary Appendix, and these have been renamed to best-case and worse-case in the appendix for consistency. (S1 appendix)

**Results**

**- Figures 2-4: since the y-axis goes below 0, I would recommend moving the x-axis labels down to align better with the end of the figure, this way the negative y-space is more visible. I would also make all y-axes the same across figures (in Figure 2, Peru panel goes to 50%, while the others stop at 40%; Figure 4 Mozambique and Peru panels go to 40% and 30% while the other stop at 20%).**

We agree, and have adjusted axes accordingly to improve clarity of Figures 2-4.

**- Struggling to understand Figure 5, and I wish it were explained a little better in the text. Are all scenarios (best, worst, catch-up and status quo) included within the colored areas of each country line? Would it be possible to maybe add dashed lines for the mean/median values of the different scenarios, or would these overlap too much? I think seeing the status quo at least separate from the disruption scenarios may be beneficial, especially for countries like Peru, Kyrgyzstan and Mozambique which seem to be significantly far from the END TB targets.**

We did try more lines and it made it less clear, but we have added additional description of the lower bound (which corresponds directly to the status quo) and the upper bound shown in Figure 5 to the text (lines 238-244).

**Appendix:**

**- I don’t understand the final time periods in the different tables: why is estimated low from Q3 2021 to Q2 2022, but estimated severe Q3 2021 to Q4 2022? Also, the "scenario" column doesn’t have the same names as Table 1, which makes the data tables a little harder to interpret - consistency between this table and the main text would be helpful.**

In line with Table 1, the best-case scenario includes a duration of disruption until 30 June 2022, while the worse-case scenario includes a duration of disruption until 31 December 2022, with each scenario including a return to status quo after that duration. An extra row for each table has been added for the best-case scenario for Q3 2022 – Q4 2022 to more clearly indicate this return to status quo and ensure all time periods line up.

The scenario column has been renamed to best-case and worse-case in the appendix for consistency. (S1 appendix)
